# Supplementary material for: Classification of ECG signal using FFT based improved Alexnet classifier
Source: PLoS One. 2022 Sep 27;17(9):e0274225. doi: 10.1371/journal.pone.0274225 (PMC9514660; doi:10.1371/journal.pone.0274225)
Supplement: Attachment — Submitted filename: Review_PLOS2.docx [file pone.0274225.s001.docx]

1. Kindly write the abstract in a concise and succinct manner, there is no requirement of background basic study in the abstract. The first three to four lines can shift to the introduction.
2. Use convolutional neural network short term in abstract.
3. Why authors are concentrated on only four beats, any specific reason?
4. Better to use any customized network not transfer learning
5. Figure 2 is copied from the internet
6. I didn’t find any comparison of the proposed method with existing literature
7. Write specific author contributions
8. MIT-BIH full form missed
9. Please remove unnecessary capitalizations in the manuscript
10. Please mention important major contributions only
11. Quality of the images are very poor, and not visible also
12. As per my knowledge authors are used Python for implementation, but network diagram missed?
13. Needs to include ROC curves, and precision-recall curves
14. Grammatically needs to recheck again
15. **Needs to recheck the structure of the paper as Introduction, Literature and Motivation, Contributions of the proposed work, Database, Proposed methodology, Experimental results, Discussion, Conclusion + Future scope.**
16. Needs to include ablation study (If possible)
17. Please cite recent scripts as below
18. Statistical tests are also missed
19. Conclusions also needs to re-write again

```
@article{prakash202113,
title={13 An Automated Diagnosis},
author={Prakash, Allam Jaya and Samantray, Saunak and Bala, CH Laxmi and Narayana, YV},
journal={Analysis of Medical Modalities for Improved Diagnosis in Modern Healthcare},
pages={301},
year={2021},
publisher={CRC Press}
}

@inproceedings{prakash2019aami,
title={AAMI standard cardiac arrhythmia detection with random forest using mixed features},
author={Prakash, Allam Jaya and Ari, Samit},
booktitle={2019 IEEE 16th India Council International Conference (INDICON)},
pages={1--4},
year={2019},
organization={IEEE}
}

@article{allam2020spec,
title={SpEC: A system for patient specific ECG beat classification using deep residual network},
author={Allam, Jaya Prakash and Samantray, Saunak and Ari, Samit},
journal={Biocybernetics and Biomedical Engineering},
volume={40},
number={4},
pages={1446--1457},
year={2020},
publisher={Elsevier}
}

@incollection{prakash2019system,
title={A system for automatic cardiac arrhythmia recognition using electrocardiogram signal},
author={Prakash, Allam Jaya and Ari, Samit},
booktitle={Bioelectronics and Medical Devices},
pages={891--911},
year={2019},
publisher={Elsevier}
}


@article{hammad2021resnet,
  title={ResNet-Attention model for human authentication using ECG signals},
  author={Hammad, Mohamed and P{\l}awiak, Pawe{\l} and Wang, Kuanquan and Acharya, Udyavara Rajendra},
  journal={Expert Systems},
  volume={38},
  number={6},
  pages={e12547},
  year={2021},
  publisher={Wiley Online Library}
}


@article{tuncer2019automated,
  title={Automated arrhythmia detection using novel hexadecimal local pattern and multilevel wavelet transform with ECG signals},
  author={Tuncer, Turker and Dogan, Sengul and P{\l}awiak, Pawe{\l} and Acharya, U Rajendra},
  journal={Knowledge-Based Systems},
  volume={186},
  pages={104923},
  year={2019},
  publisher={Elsevier}
}

@article{ksikazek2021comparison,
  title={Comparison of various approaches to combine logistic regression with genetic algorithms in survival prediction of hepatocellular carcinoma},
  author={Ksi{\k{a}}{\.z}ek, Wojciech and Gandor, Micha{\l} and P{\l}awiak, Pawe{\l}},
  journal={Computers in Biology and Medicine},
  volume={134},
  pages={104431},
  year={2021},
  publisher={Elsevier}
}
```
