# Supplementary material for: Classification of ECG signal using FFT based improved Alexnet classifier
Source: PLoS One. 2022 Sep 27;17(9):e0274225. doi: 10.1371/journal.pone.0274225 (PMC9514660; doi:10.1371/journal.pone.0274225)
Supplement: Attachment — Submitted filename: Response to reviewer comments Ver 2.0.docx [file pone.0274225.s003.docx]

**Reviewer #1:**

Authors are incorporated all my comments. Only two minor suggestions from my side.

**Comment 1:** Figure 2 should be changed; the author can generate one ECG beat segment using MATLAB or Python.

**Response:** Thank you for valuable suggestion. As per the comment, one ECG beat segment is generated using python and the same is included in the revised manuscript.

*Figure 2: ECG beat signal*

**Comment 2:** The copied figure from the internet should be replaced with a new figure before publication.

**Response:** Figure 2 is replaced with the new one in the revised paper.
